# HDAC4 regulates satellite cell proliferation and differentiation by targeting P21 and Sharp1 genes

**DOI:** 10.1038/s41598-018-21835-7

**Published:** 2018-02-22

**Authors:** Nicoletta Marroncelli, Marzia Bianchi, Marco Bertin, Silvia Consalvi, Valentina Saccone, Marco De Bardi, Pier Lorenzo Puri, Daniela Palacios, Sergio Adamo, Viviana Moresi

**Affiliations:** 1 DAHFMO Unit of Histology and Medical Embryology, Interuniversity Institute of Myology, Sapienza University of Rome, Rome, 00161 Italy; 2 IRCCS Fondazione Santa Lucia, Via del Fosso di Fiorano, 64, Rome, 00143 Italy; 3 Laboratory of Cardiovascular Endocrinology, IRCCS San Raffaele Pisana, Rome, 00166 Italy

## Abstract

Skeletal muscle exhibits a high regenerative capacity, mainly due to the ability of satellite cells to replicate and differentiate in response to appropriate stimuli. Epigenetic control is effective at different stages of this process. It has been shown that the chromatin-remodeling factor HDAC4 is able to regulate satellite cell proliferation and commitment. However, its molecular targets are still uncovered. To explain the signaling pathways regulated by HDAC4 in satellite cells, we generated tamoxifen-inducible mice with conditional inactivation of HDAC4 in Pax7^+^ cells (HDAC4 KO mice). We found that the proliferation and differentiation of HDAC4 KO satellite cells were compromised, although similar amounts of satellite cells were found in mice. Moreover, we found that the inhibition of HDAC4 in satellite cells was sufficient to block the differentiation process. By RNA-sequencing analysis we identified P21 and Sharp1 as HDAC4 target genes. Reducing the expression of these target genes in HDAC4 KO satellite cells, we also defined the molecular pathways regulated by HDAC4 in the epigenetic control of satellite cell expansion and fusion.

## Introduction

Skeletal muscle integrity and homeostasis largely depend on its striking capacity to regenerate after damage or upon physiological requests, such as growth or exercise. Muscle regeneration mainly relies on a specific type of muscle stem cells, the satellite cells. Upon appropriate stimulation, satellite cells exit quiescence, proliferate and differentiate into mature myofibers. Sequential expression of myogenic regulatory factors (MRFs) and epigenetic regulators are crucial factors in satellite cell expansion and commitment^1,2^.

The basic helix-loop-helix transcription factor MyoD is an important regulator of myogenic differentiation^3^. The ectopic expression of MyoD stimulates the conversion of different cell lines into skeletal muscle^4^. Although MyoD mutant mice do not show overt abnormalities in skeletal muscle development, they are not able to regenerate efficiently after trauma. These observations suggest a role for MyoD in adult skeletal muscle regeneration^5,6^. On the one hand, MyoD triggers withdrawal from the cell cycle before the differentiation process by inducing the expression of p21Cip-1/Waf-1 (P21)^7^, a cyclin-dependent kinase inhibitor that blocks cell proliferation^8^. On the other hand, MyoD collaborates with members of the myocytes enhancer factor 2 (MEF2) family in activating muscle-specific genes and myogenesis^9^. While MyoD is expressed in proliferating myoblasts and bound to several genomic loci^10^, it is unable to activate transcription due to the epigenetic regulation of chromatin structure. Namely, HDACs and heterochromatin proteins HP1, Ezh2 and Suv39h1 orchestrate histone deacetylation and methylation, repressing MyoD-dependent muscle gene transcription^11–16^. In addition, Sharp1 cooperates with G9a on the inhibition of myogenic differentiation by modulating histone and MyoD methylation^17,18^.

Several epigenetic mechanisms regulate the sequential activation of myogenic factors. Alterations in the epigenetic pathways are associated with muscle disorders and may influence them^1,19^. Quiescent satellite cells are characterized by an open and permissive chromatin state and are primed for activation and differentiation in response to appropriate external stimuli. At the chromatin level, the primed state is maintained by the presence of the H3K4me3 mark at the transcription start sites of a large number of genes, including MRFs such as MyoD^20–22^. In addition, the genes that control differentiation programs often harbor bivalent chromatin domains, which are characterized by a combination of H3K4me3 and H3K27me3 marks^23^, keeping stem cells primed. Myogenic differentiation is associated with gene repression and characterized by an increase in repressive histone marks^21,24^. The acetylation state of histones also contributes to chromatin remodeling. Two families of antagonistic enzymes, histone acetyltransferases (HATs) and histone deacetylases (HDACs), catalyze the acetylation and the deacetylation of histones, acting as transcriptional activators and repressors, respectively. As epigenetic regulators, HATs and HDACs control satellite cell differentiation. In undifferentiated muscle cells, class I HDACs repress MyoD activity, whereas members of class II HDACs associate with MEF2 and block its activity, thus inhibiting muscle cell differentiation. During differentiation, the formation of a pRb-HDAC1 complex induces the disruption of the MyoD–HDAC1 complex and the transcriptional activation of the differentiation genes^25^. Moreover, increasing levels of MRFs and MEF2 factors overcome the capacity of class II HDACs to repress MEF2-dependent genes, inducing muscle differentiation^26^. Hypertrophic and differentiation stimuli induce the nuclear-cytoplasmic shuttling of HDAC4 and its dissociation from MEF2 factors, promoting muscle growth^26^. Several kinases are able to phosphorylate class II HDAC members in response to different stimuli, including calcium/calmodulin dependent kinase (CaMK), extracellular signal-regulated MAP kinase (ERK1/2), protein kinase A (PKA) or glycogen-synthase kinase 3 (GSK3), inducing the localization of class II HDAC to the cytoplasm^27^. Conversely, reverse translocation is regulated by phosphatase 2 A, which dephosphorylates the residues recognized by 14-3-3 proteins^28^. Among class II HDACs, HDAC4 seems to mediate cellular responses to environmental perturbations, including denervation and muscle injury^29–32^. However, the underlying molecular mechanisms remain unclear.

Here, we report the identification of two molecular targets of HDAC4 in satellite cells. Through these target genes, HDAC4 regulates the gene networks associated with cell proliferation and differentiation. In particular, we show that HDAC4-mediated repression of the cell cycle inhibitor P21 promotes satellite cell amplification, while the repression of the basic helix-loop-helix transcription factor Sharp1 allows satellite cell differentiation and fusion. These data suggest that HDAC4 regulates the genes that control two sequential stages of satellite cell activity during myogenesis, that is proliferation and differentiation into new fibers.

## Results

### HDAC4 is required for satellite cell differentiation

**Figure 1:**
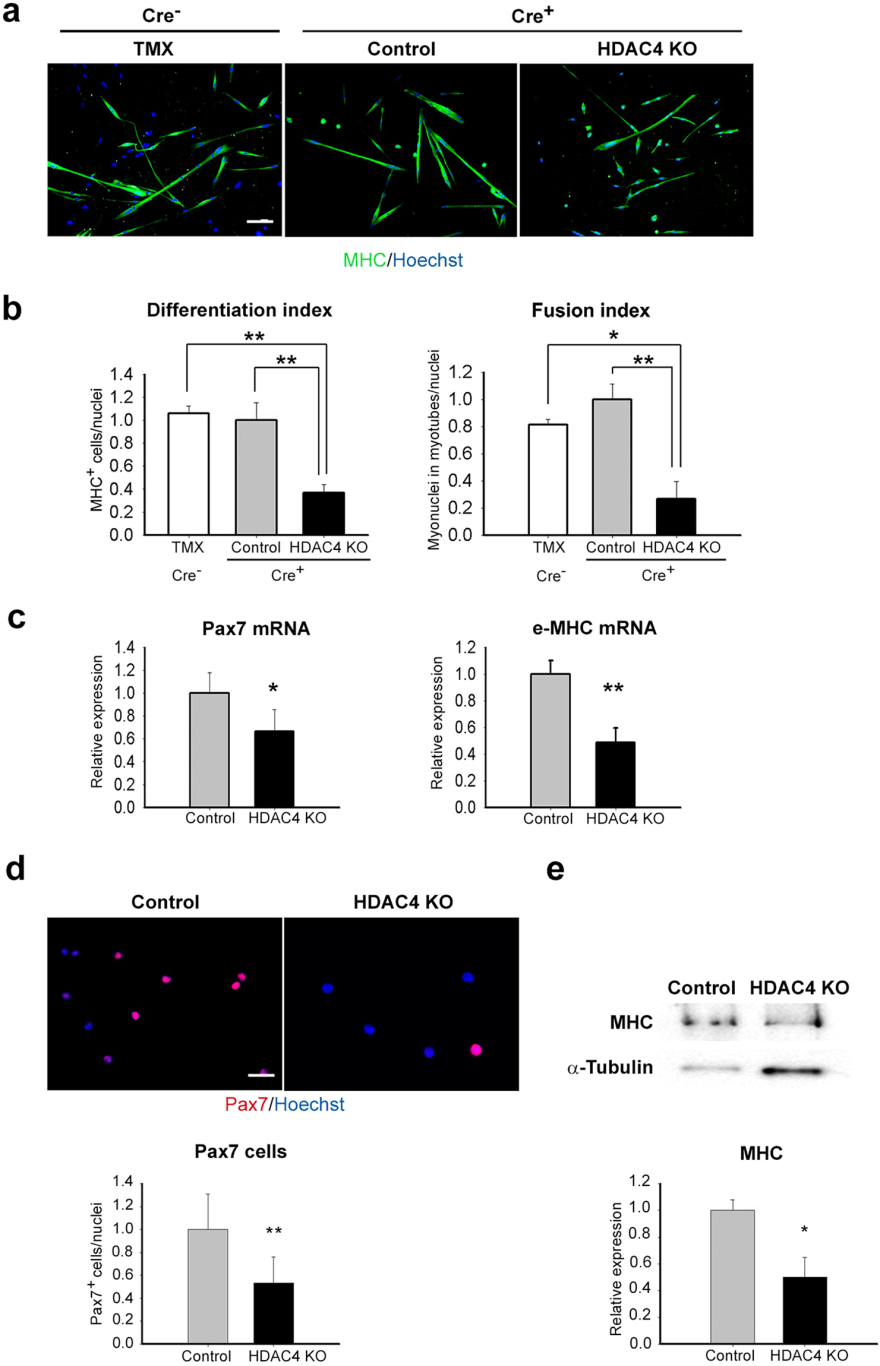
HDAC4 is essential for satellite cell differentiation. (**a**) Immunofluorescence for MHC in satellite cells derived from HDAC4^fl/fl^ mice after TMX injections (Cre^−^ TMX) and from HDAC4^fl/fl^ Pax7^CE^ mice treated with TMX (HDAC4 KO) or vehicle (Control), after three days in differentiation medium. Scale bar: 100 μm. (**b**) Quantification of the differentiation and fusion indexes in Cre^−^ TMX, HDAC4 KO and control satellite cells. n = 6 per condition. Data are presented as mean ± SEM. *p < 0.05; **p < 0.005 (Student’s t-test). (**c**) Expression levels of myogenic markers in HDAC4 KO satellite cells, compared to the controls, by real time PCR. n = 5 each sample. Data are presented as mean ± SEM. *p < 0.05; **p < 0.005 (Student’s t-test). (**d**) Representative pictures of immunofluorescence analysis for Pax7 and relative quantification in HDAC4 KO and control satellite cells, in growth conditions. Scale bar: 50 μm. n = 10 per genotype. Data are presented as mean ± SEM. **p < 0.005 (Student’s t-test). (**e**) Representative western blot (cropped blot) with relative densitometry of MHC (n = 3 per sample) in HDAC4 KO satellite cells, compared to the controls. α-Tubulin was used as a loading control. Full-length blot is presented in Supplementary Figure S2b. Data are expressed relative to control mice as average +/− SEM. n = 3 per genotype. *p < 0.05 (Student’s t-test).

A global deletion of HDAC4 is associated with early lethality in mice^33^. Therefore, in order to investigate the role of HDAC4 in satellite cells, we crossed HDAC4^fl/fl^ mice with a transgenic mouse line expressing a tamoxifen (TMX)-inducible Cre recombinase-Estrogen Receptor fusion protein, Cre-ERT2, under the control of Pax7 promoter (Pax7^CE^)^34,35^. It should be noted that Cre recombinase was inserted into the Pax7 gene, hence Pax7^CE^ mice expressed only one allele of Pax7. For this reason, we only used Pax7^CE^ mice in our experiments and treated them with TMX (hereafter referred to as HDAC4 KO mice) or vehicle, as control mice. To evaluate the effects of TMX on satellite cells, we also analysed the satellite cells from HDAC4^fl/fl^ mice after TMX injections (Cre^−^ TMX), as additional controls in the first experiment. After 5 days of intraperitoneal injections, satellite cells were isolated according to the differential expression of surface markers by fluorescence-activated cell sorting (FACS) or by MACS Microbeads technology^36^. Real-time PCR and western blot analyses confirmed a significant decrease in HDAC4 expression in HDAC4 KO satellite cells compared to controls (Supplementary Figs S1a,b and S2a). To test if HDAC4 affects satellite differentiation, satellite cells were induced to differentiate, and terminal differentiation was assessed by immunofluorescence (IF) analyses for Myosin Heavy Chain (MHC) (Fig. 1a). HDAC4 deletion in satellite cells resulted in impaired differentiation, as quantified by differentiation index, i.e. the number of myonuclei in MHC positive cells over the total nuclei, and fusion index, the number of myonuclei in myotubes over the total nuclei (Fig. 1b). Conversely, no significant differences were detected between Cre^−^ TMX and controls (Fig. 1a,b), indicating that TMX treatment did not affect satellite cell differentiation. Consistently, molecular analyses by real-time PCR showed lower expression of the myogenic markers Pax7 and embryonic MHC (e-MHC) in HDAC4 KO satellite cells (Fig. 1c). Moreover, immunofluorescence for Pax7 and western blot analyses for MHC confirmed a significant decrease in these myogenic markers in HDAC4 KO satellite cells compared with controls (Fig. 1d,e and Supplementary Fig. S2b). These data reveal that satellite cells cannot efficiently differentiate when HDAC4 deletion occurs.

### Intrinsic absence of HDAC4 inhibits satellite cell differentiation

Although satellite cells are the direct effectors of skeletal muscle repair following injury, their behavior is modulated by the factors secreted by many other cell types^37–44^. To assess if HDAC4 affects satellite cell differentiation through soluble factors, we cultured wild-type satellite cells with conditioned media from control or HDAC4 KO satellite cells. No differences between the experimental conditions were observed (Supplementary Fig. S3a,b).

**Figure 2:**
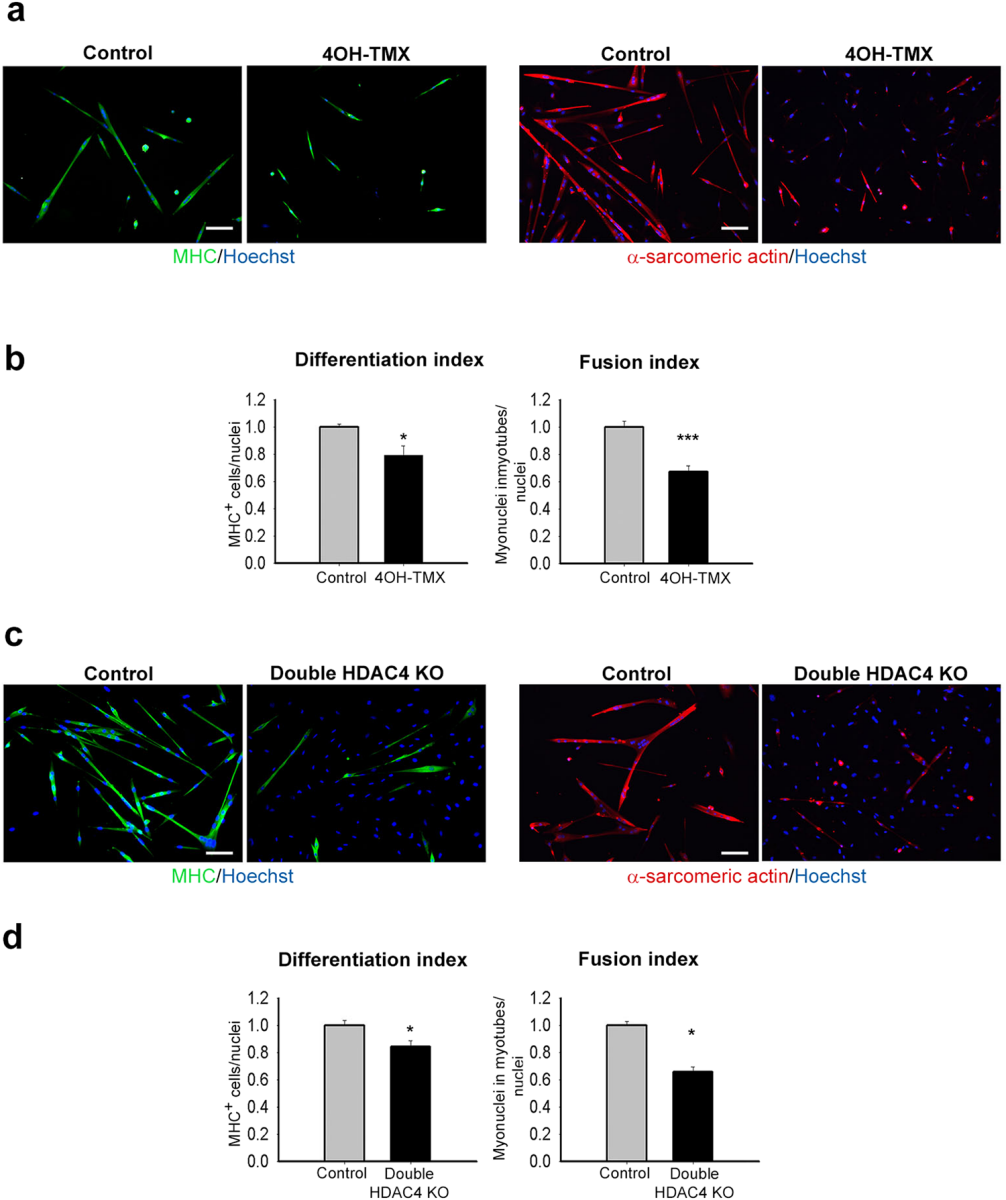
Deletion of HDAC4 in satellite cells is sufficient to inhibit differentiation. (**a**) Immunofluorescences for MHC and α-sarcomeric actin in satellite cells from HDAC4^fl/fl^ Pax7^CE^ mice treated with 4OH-TMX or vehicle, after three days in differentiation medium. Scale bar: 100 μm. (**b**) Quantification of the differentiation and fusion indexes. n = 4 each sample. Data are presented as mean ± SEM. *p < 0.05; ***p < 0.001 (Student’s t-test). (**c**) Immunofluorescence for MHC and α-sarcomeric actin in HDAC4 KO plated at double density and control satellite cells, after three days in differentiation medium. Scale bar: 100 μm. (**d**) Quantification of the differentiation and fusion indexes. n = 3 each sample. Data are presented as mean ± SEM. *p < 0.05 (Student’s t-test).

Moreover, skeletal muscle niche modulates satellite cell behaviour *in vivo*^45^. To test any effect of *in vivo* microenvironment on HDAC4 KO satellite cells, we sorted satellite cells from Pax7^CE^ mice and treated them for 48 hours with 4OH-TMX, the active metabolite of TMX, or vehicle as controls *in vitro*. HDAC4 expression was significantly reduced in Pax7^CE^ cells treated with 4OH-TMX as shown by real-time PCR (Supplementary Fig. S3c). Satellite cell differentiation was assessed by IF for MHC and α-sarcomeric actin, after three days in differentiation medium. Quantification of the differentiation and fusion indexes demonstrated that HDAC4 excision impaired satellite cell terminal differentiation (Fig. 2a,b).

Satellite cell differentiation is triggered by cell-cell contact^46^. To test whether the impaired differentiation of HDAC4 KO cells in culture was a consequence of a lower density, we plated HDAC4 KO satellite cells at double density compared to controls, and evaluated cell differentiation by IF for MHC and α-sarcomeric actin. Still, HDAC4 KO satellite cells displayed impaired differentiation, as shown by IF analyses and quantification of the differentiation and fusion indexes (Fig. 2c,d).

### HDAC4 regulates satellite cell proliferation

**Figure 3:**
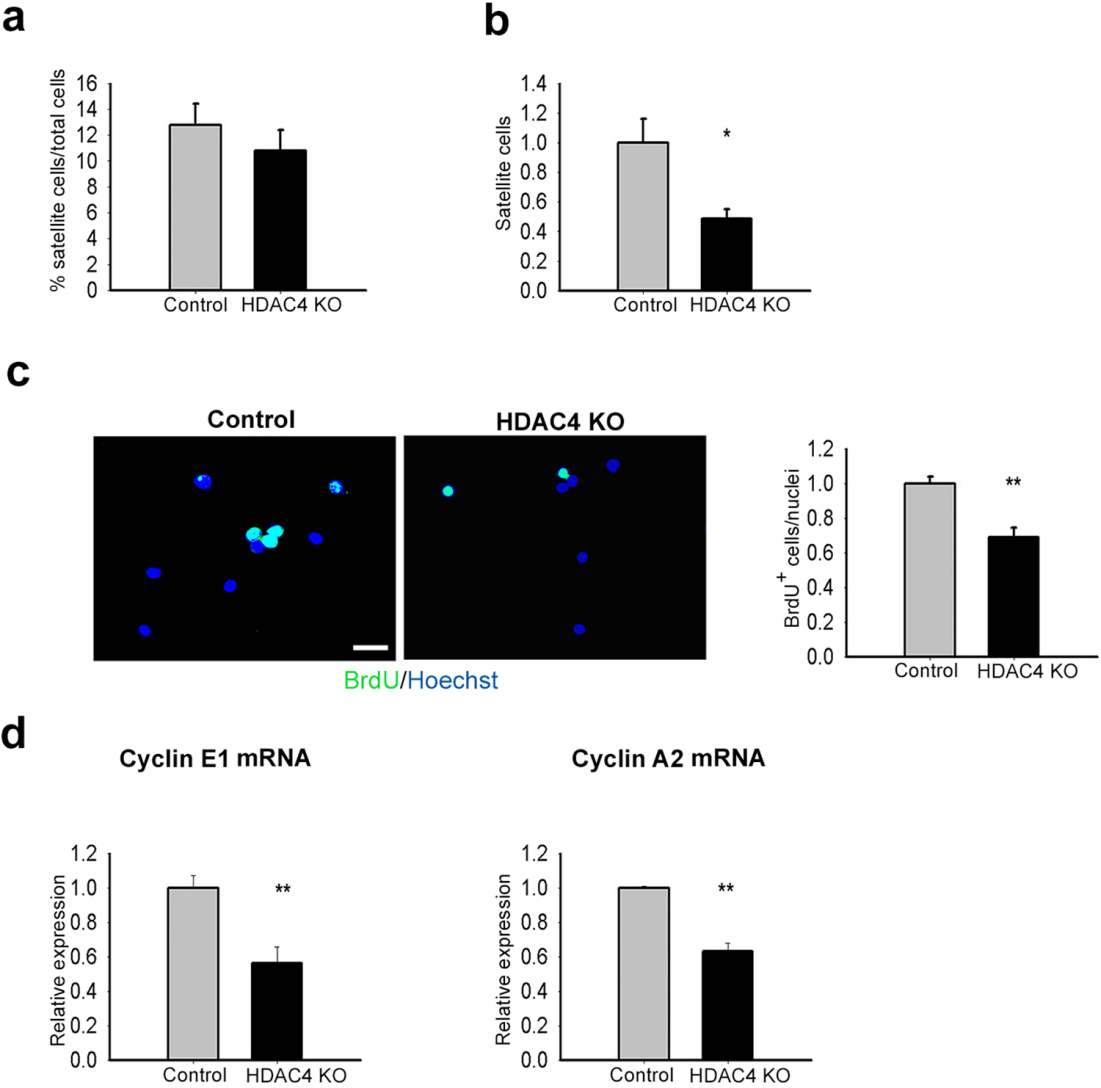
HDAC4 affects satellite cell proliferation. (**a**) Percentage of satellite cell population isolated from control and HDAC4 KO mice by FACS sorting. n = 8 for each genotype. Data are presented as mean ± SEM. (**b**) Number of satellite cells after two days in growing conditions, compared to controls. n = 5 each sample. Data are presented as mean ± SEM. *p < 0.05 (Student’s t-test). (**c**) BrdU assay and quantification in control and HDAC4 KO samples. Scale bar: 50 μm. n = 5 for genotype. Data are presented as mean ± SEM. **p < 0.005 (Student’s t-test). (**d**) Expression of proliferation markers in control and HDAC4 KO satellite cells, by real-time PCR. n = 4 each sample. Data are presented as mean ± SEM. **p < 0.005 (Student’s t-test).

The number of satellite cells isolated from HDAC4 KO muscles, over total cells, was not significantly different from that of control mice (Fig. 3a). However, after two days in culture, we observed and quantified a lower number of satellite cells in HDAC4 KO samples compared with those in the controls (Fig. 3b). To investigate if the reduction in satellite cell number was caused by a decrease in proliferation, satellite cells were pulse-labelled with the thymidine analogue 5-bromo-2′-deoxyuridine (BrdU), which allows the detection of newly synthesized DNA. The number of BrdU^+^ cells was counted and normalized to total cells, showing that HDAC4 KO satellite cells proliferated significantly less than control cells (Fig. 3c). Furthermore, deletion of HDAC4 in satellite cells significantly down-regulated the expression of proliferation markers cyclin E1 and cyclin A2, compared to the control levels (Fig. 3d). No significant differences were detected in apoptosis between control and HDAC4 KO satellite cells, as assessed by a Tunel assay and the evaluation of the expression of apoptotic markers Bax and Bcl-2 (Supplementary Fig. S4a,b).

### Deletion of HDAC4 in satellite cells compromises muscle regeneration

**Figure 4:**
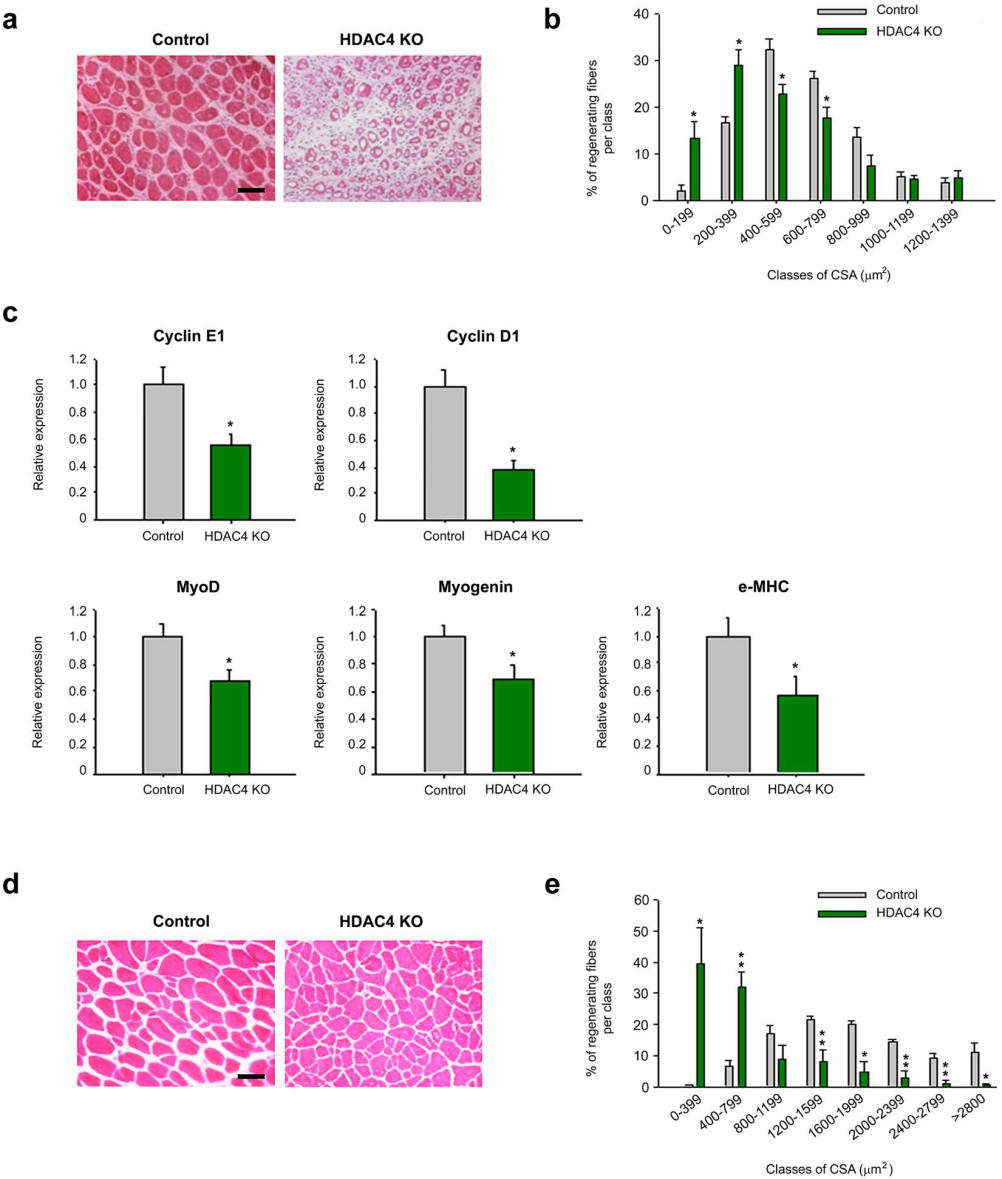
HDAC4 is essential for skeletal muscle regeneration. (**a**) Representative images of tibialis anterior regenerating muscles, one week after injury. Scale bar: 50 μm. (**b**) Morphometric analysis of the distribution of regenerating fiber cross-sectional area, one week after injury. n = 5. Data are presented as average +/− SEM. *p < 0.05 by Student’s t test. (**c**) Real-time PCR for proliferative and myogenic markers in HDAC4 KO and control mice, 2.5 days following injury. n = 7. Data are presented as average +/− SEM. *p < 0.05 (Student’s t-test). (**d**) Representative images of TA regenerating muscles, one month after injury. Scale bar: 50 μm. (**e**) Morphometric analysis of the distribution of regenerating fiber cross-sectional area, one month after injury. n = 5. Data are presented as average +/−SEM. *p < 0.05; **p < 0.005 (Student’s t-test).

To confirm the role of HDAC4 in satellite cells *in vivo*, we evaluated muscle regeneration in HDAC4 KO and control mice by morphometrical and molecular analyses. To induce muscle regeneration, tibialis anterior muscles of adult mice were subjected to a localized, reproducible freeze injury^47^. One week after injury, histological analyses revealed that regenerating, centronucleated, myofibers were smaller in HDAC4 KO mice compared to controls and interspersed among mononuclear infiltrating cells (Fig. 4a). Morphometric analyses of regenerating myofibers confirmed that in HDAC4 KO mice smaller regenerating fibers (0–399 μm^2^) significantly outnumbered the bigger ones (400–799 μm^2^) (Fig. 4b). Compromised cell proliferation and myogenic differentiation were also assessed by molecular analyses of proliferative (cyclin E1 and cyclin D1) and myogenic (MyoD, myogenin and e-MHC) markers in HDAC4 KO muscles, 2.5 days after injury (Fig. 4c).

To investigate whether muscle regeneration was delayed or severely compromised in HDAC4 KO mice, we performed histological evaluation of skeletal muscles one month following injury. HDAC4 KO mice displayed smaller regenerating fibers compared with controls, even one month after injury (Fig. 4d). Morphometric analyses confirmed that HDAC4 KO mice displayed a higher number of smaller regenerating myofibers (0–799 μm^2^) than control mice, at the expenses of the bigger ones (1200–3000 μm^2^) (Fig. 4e).

### Identification of HDAC4 direct targets involved in satellite cell proliferation and differentiation

**Figure 5:**
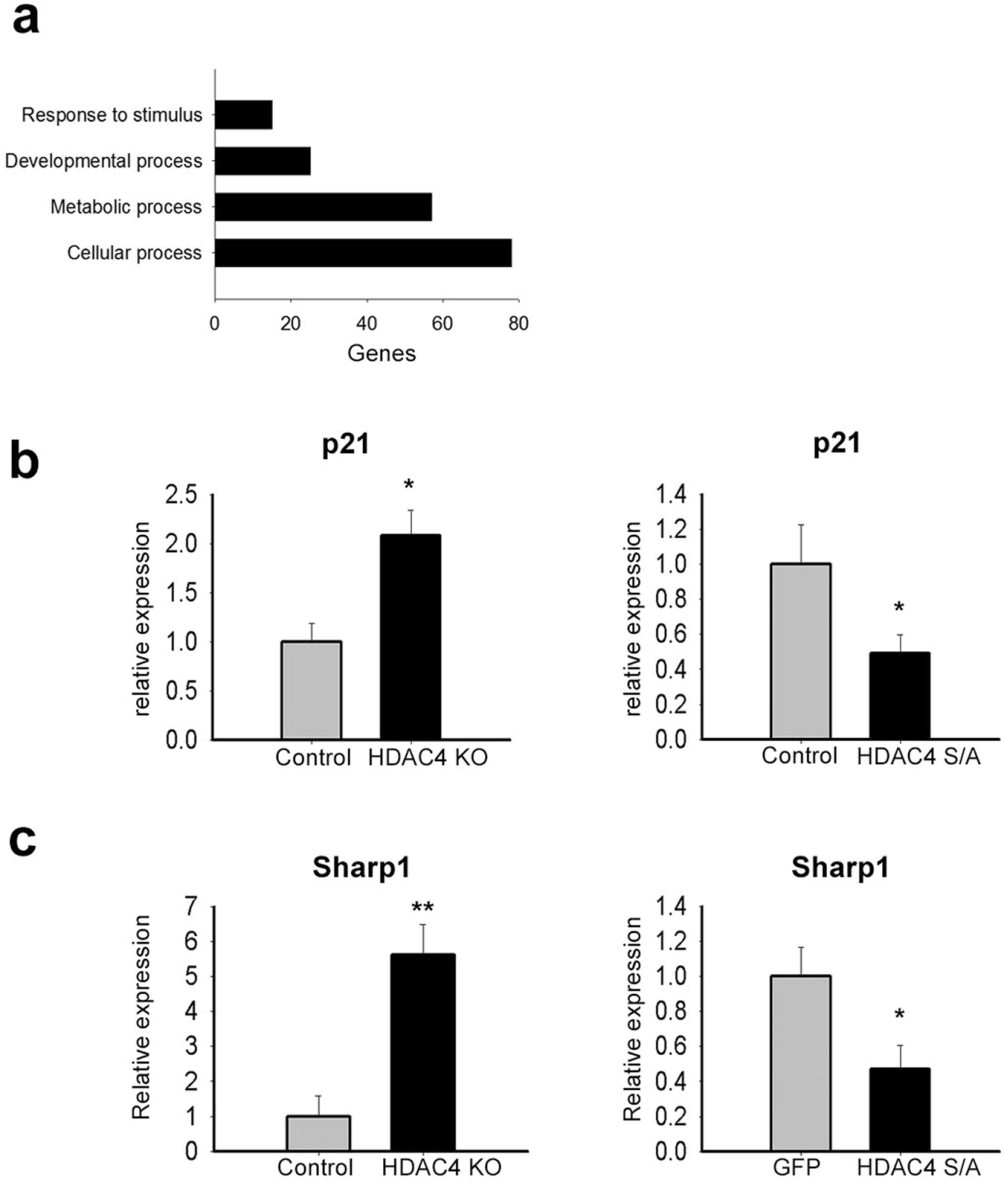
Identification of HDAC4 target genes. (**a**) Gene ontology analyses on 1231 genes differentially modulated by HDAC4 in satellite cell proliferation. (**b**) Expression of P21 in HDAC4 KO and control satellite cells (n = 4 each sample) or in primary myotubes overexpressing the nuclear form of HDAC4 (HDAC4 S/A), compared to GFP-expressing myotubes (n = 5 each sample). Data are presented as mean ± SEM. *p < 0.05 (Student’s t-test). (**c**) Expression of Sharp1 in HDAC4 KO and control satellite cells (n = 4 each sample) or in primary myotubes overexpressing the nuclear form of HDAC4 (HDAC4 S/A), compared to GFP-expressing myotubes (n = 5 each sample). Data are presented as mean ± SEM. *p < 0.05; **p < 0.005 (Student’s t-test).

To identify the genes modulated by HDAC4 in proliferating satellite cells, we performed a transcriptome analysis of control and HDAC4 KO satellite cells. Total RNA was isolated from duplicates of HDAC4 KO and control satellite cells after 24 hours in growth conditions, and samples were subjected to RNA sequencing (RNA-seq) analysis. This early time point was used to select direct HDAC4 targets upon satellite cells activation. Significant changes in gene expression only occurred in a small number of genes in HDAC4 KO cells. Therefore, we used a cut-off value of 1.2 for gene expression fold change. A total of 1263 genes were significantly modulated in the absence of HDAC4 (p value < 0.05). In particular, we found 807 up-regulated and 456 down-regulated genes compared to control samples. Gene ontology analysis revealed that, among the biological processes, HDAC4 mostly affected cellular processes (Fig. 5a and Supplementary Table S1) including cell cycle, in line with the HDAC4 KO cell phenotype.

Searching for HDAC4 targets that may be involved in the regulation of cell proliferation, we identified P21 as an attractive candidate from the list of up-regulated genes in HDAC4 KO muscle cells, due to its pivotal role in muscle cell proliferation^8^. We confirmed that P21 was significantly up-regulated in HDAC4 KO cells, whereas it was down-regulated in the myotubes over-expressing a nonphosphorylatable mutant form of HDAC4, HDAC4 S/A, which is specifically retained in the nucleus^48^ (Fig. 5b and Supplementary Fig. S5).

In addition, among the list of up-regulated genes in HDAC4 KO cells, we focused on Sharp1, since it is known to be involved in the control of muscle cell differentiation^49,50^. More importantly, Sharp1 expression inversely correlated with that of HDAC4 in HDAC4 KO satellite cells and in myotubes over-expressing HDAC4 S/A (Fig. 5c).

### P21 and Sharp1 mediate HDAC4-depentent inhibition of satellite cell proliferation and differentiation

**Figure 6:**
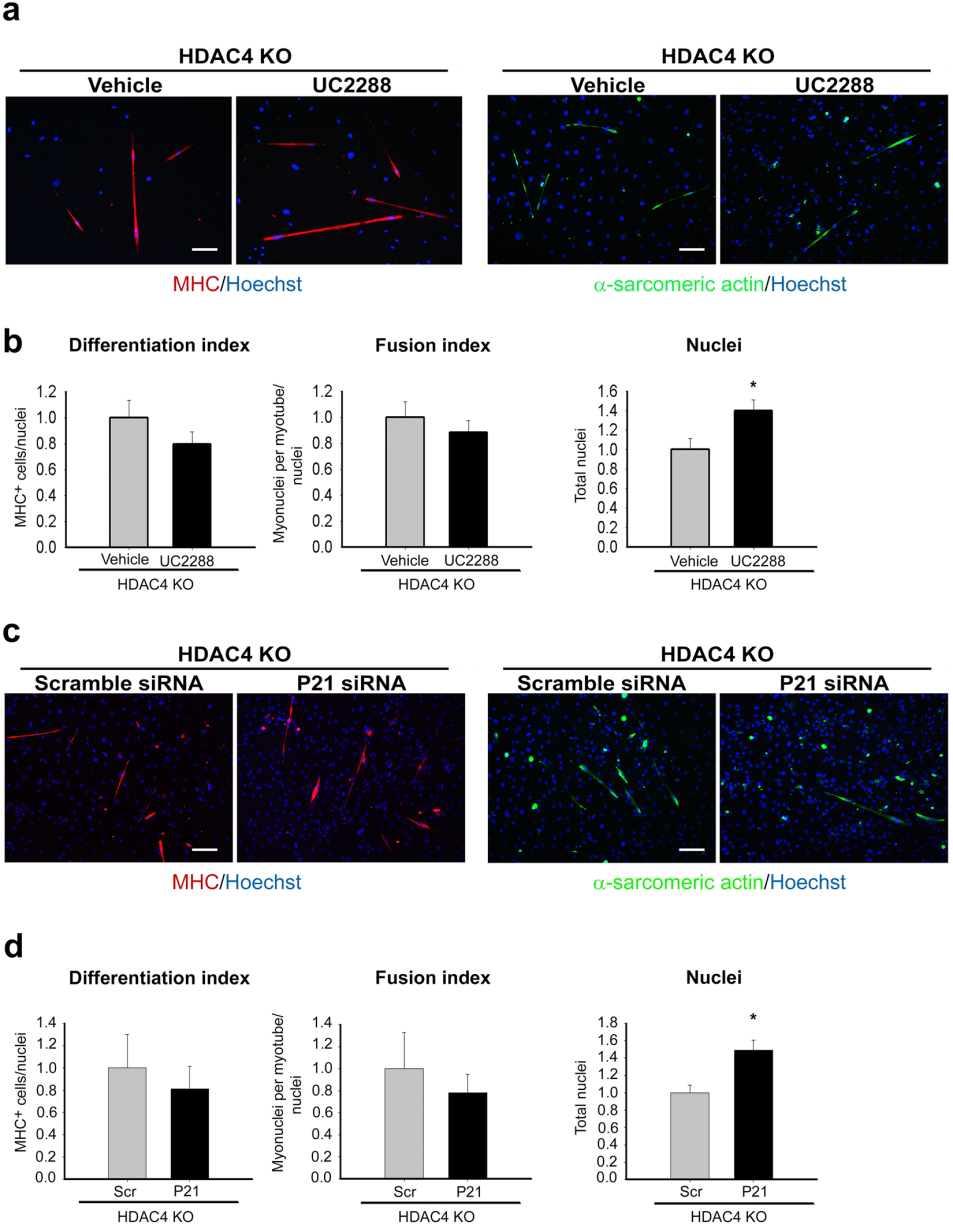
P21 is a HDAC4 target gene responsible for satellite cell proliferation. (**a**) Immunofluorescence for MHC and α-sarcomeric actin in HDAC4 KO satellite cells treated with UC2288 or vehicle for 24 hours and differentiated for three days. Scale bar: 100 μm. (**b**) Quantification of the differentiation and fusion indexes; counts of total nuclei relative to vehicle-treated samples. n = 4 per condition. Data are presented as mean ± SEM. *p < 0.05 (Student’s t-test). (**c**) Immunofluorescence for MHC and α-sarcomeric actin in HDAC4 KO satellite cells transfected with a pool of four siRNA against P21 (P21 siRNA) or a pool of non-targeting siRNA (Scramble siRNA), as control and differentiated for three days. Scale bar: 100 μm. (**d**) Quantification of the differentiation and fusion indexes; counts of total nuclei relative to Scramble (Scr)-treated samples. n = 4 per condition. Data are presented as mean ± SEM. *p < 0.05 (Student’s t-test).

To functionally validate the role of P21 in HDAC4 KO satellite cell proliferation, we took advantage of the pharmacological inhibitor, UC2288^51,52^, which efficiently down-regulates P21 levels in HDAC4 KO satellite cells (Supplementary Fig. S6a). HDAC4 KO satellite cells were treated for 24 hours with 10 µM UC2288, or vehicle as control, in growth condition. Then, the cultures were shifted to differentiation conditions. After three days, satellite cell differentiation was evaluated by MHC and α-sarcomeric actin staining (Fig. 6a). Quantification of the differentiation and fusion indexes revealed that inhibition of P21 did not increase differentiation or fusion of HDAC4 KO cells, despite a significant increase in the number of satellite cells (Fig. 6b).

To confirm that the effects of UC2288 depended on P21 inhibition, we down-regulated P21 expression in HDAC4 KO satellite cells by using siRNA approach. HDAC4 KO satellite cells were transfected with a pool of four siRNA against P21, which efficiently down-regulate P21 expression (Supplementary Fig. S6b), and a pool of non-targeting siRNA as control. Twenty-four hours later, satellite cells were shifted to differentiation conditions. After three days, terminal differentiation was evaluated by MHC and α-sarcomeric actin staining (Fig. 6c). Quantification of the differentiation and fusion indexes and satellite cell counts confirmed the results obtained with the P21 inhibitor UC2288 (Fig. 6d).

**Figure 7:**
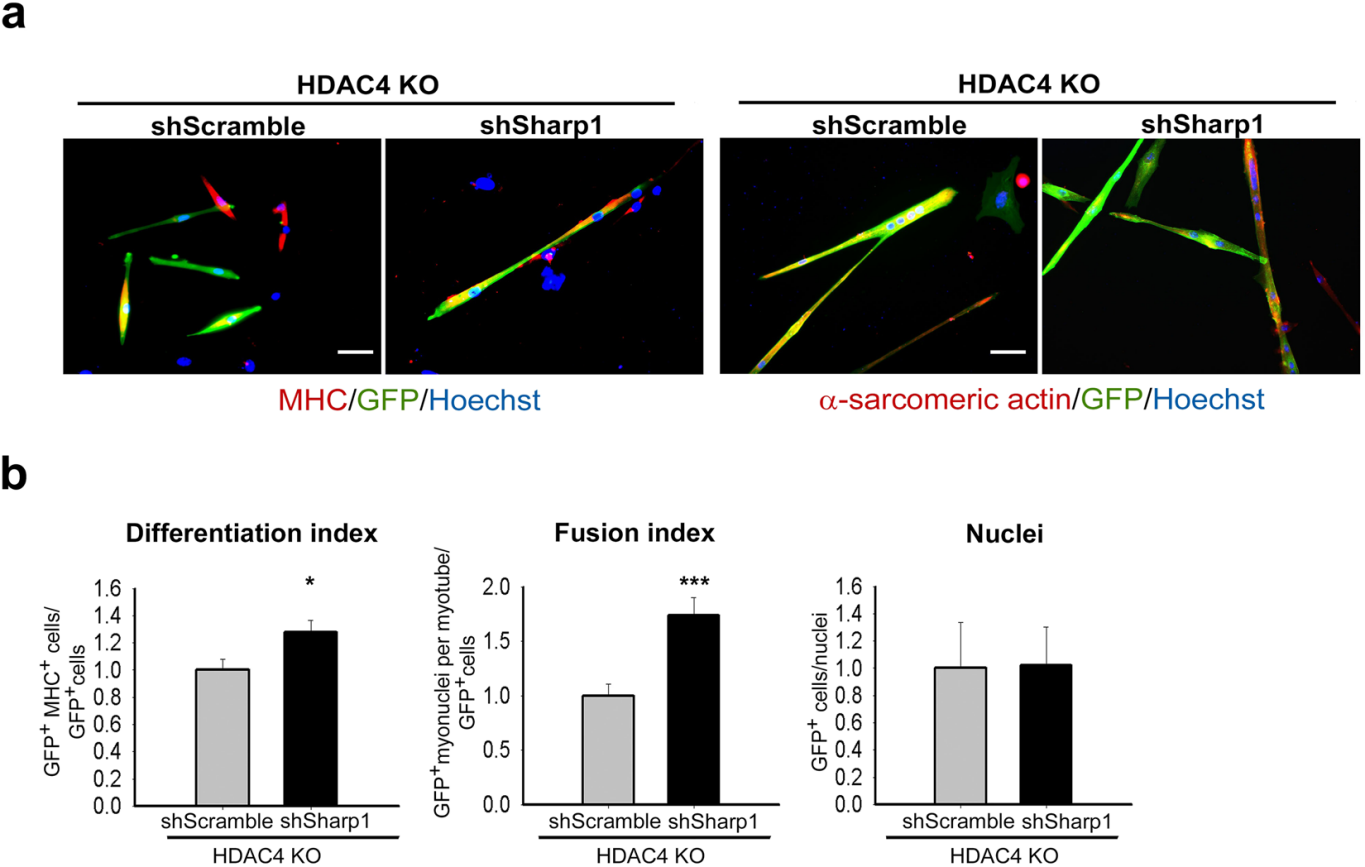
Sharp1 is a HDAC4 target gene responsible for satellite cell differentiation and fusion. (**a**) Immunofluorescence for MHC or α-sarcomeric actin and GFP in HDAC4 satellite cells transfected with scramble or Sharp1 shRNA expressing vector. Scale bar: 50 μm. (**b**) Quantification of the differentiation and fusion indexes, relative to GFP^+^ cells; count of nuclei in GFP^+^ cells, compared to total nuclei. n = 5 experiments. Data are presented as mean ± SEM. *p < 0.05; ***p < 0.001 (Student’s t-test).

To functionally validate that Sharp1 was involved in satellite cell differentiation, we down-regulated its expression in HDAC4 KO satellite cells. To this purpose, HDAC4 KO satellite cells were transfected with a shRNA against Sharp1 plasmid, which was previously tested on muscle cells for its ability to down-regulate Sharp1 expression (Supplementary Fig. S7), or shRNA control plasmid DNA, in combination with a GFP-expressing plasmid, to identify the transfected cells. Terminal differentiation was assessed by MHC and α-sarcomeric actin staining (Fig. 7a). Morphometric analyses revealed that inhibition of Sharp1 is sufficient to increase HDAC4 KO satellite cell differentiation and fusion, although no significant differences were recorded in the number of GFP-transfected cells (Fig. 7b).

## Discussion

Satellite cells activation, commitment and differentiation into skeletal muscle are controlled by numerous epigenetic mechanisms, including DNA methylation, histone modifications, and RNA-associated silencing^53–55^. The role of histone acetylation and deacetylation in the control of myogenesis has been studied mainly *in vitro*. Despite the fact that global histone acetylation progressively decreases during myogenesis, upon differentiation, histone acetylation increases in a subset of genes that are important for myogenic differentiation such as MyoD target genes^10,56^. So far, class I and class II HDACs have been consistently reported to act in different phases of muscle differentiation. Indeed, HDAC1 and HDAC2 are mainly involved in the replication stage, by repressing MyoD expression and activity, as well as the expression of muscle genes, such as myogenin, creatine kinase, or MEF2^22,25,57,58^. Conversely, class II HDACs are required for muscle differentiation, since HDAC4 and HDAC5 repress MEF2 activity^59^. Upon differentiation, the release of class I and class II HDACs targets has been linked to the decrease in the expression, a variation in the nucleus/cytoplasm distribution ratio, or to the formation of different complexes with partner proteins^25,26,59,60^, allowing the recruitment of chromatin-remodelling factors and the transcription of muscle genes^57,58,61^.

**Figure 8:**
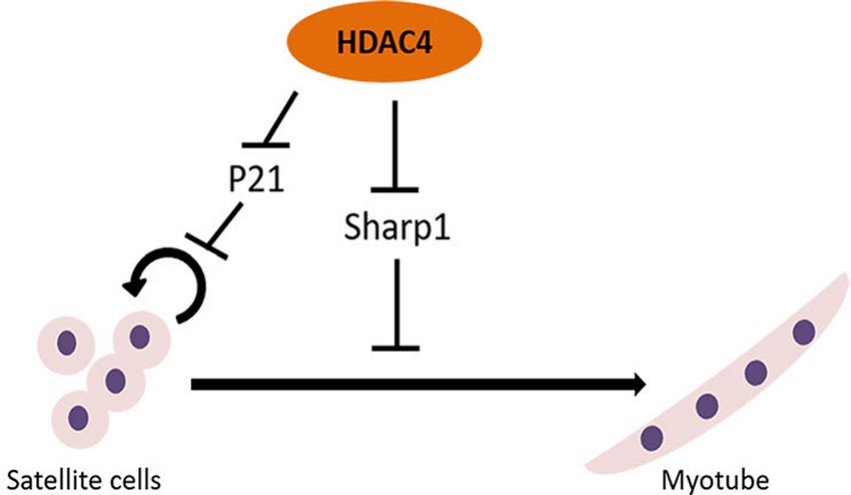
Working model of HDAC4 function in satellite cells. During proliferative stage, HDAC4 represses P21 and Sharp1 expression, thereby regulating satellite cell proliferation and differentiation.

Our study complements this model, identifying the role of HDAC4 also in the control of the proliferation phase of muscle stem cells (Fig. 8). By using an inducible, tissue-specific KO mouse line, we report that HDAC4 KO satellite cells display decreased proliferation and differentiation *in vitro* and *in vivo*, as shown also by Yao’s group^32^. Contrary to that study, in our work we only used Cre^+^ mice treated with TMX or vehicle, to overcome the monoallelic expression of Pax7 in the Cre^+^ mice, which can affect satellite cell behaviour. As additional control, in the first experiment, we also treated Cre^−^ mice with TMX, and found no effect of TMX on satellite cells.

Conditioned media from HDAC4 KO satellite cells did not affect wild-type satellite cell differentiation, suggesting that diffusible factors are not involved in this phenomenon. Furthermore, *in vitro* excision of HDAC4 through an active metabolite of TMX significantly reduced satellite cell differentiation and fusion. These results clearly indicate that the reduction in HDAC4 expression is sufficient to inhibit satellite cell differentiation, in spite of what was observed by others^32^. Yao’s group showed that siRNA knockdown of HDAC4 in purified satellite cells did not cause a reduction in Pax7 expression, which led the authors to conclude that regulation of Pax7 by HDAC4 is indirect and requires an intact muscle microenvironment *in vivo*. However, the ability of siRNA-treated satellite cells to differentiate was not shown. Conversely, we found that 4-OH TMX-treated satellite cells showed similar levels of Pax7 over the control (data not shown), suggesting that HDAC4 regulates satellite cell proliferation and differentiation through other molecular targets.

To identify the pathways affected by HDAC4 in satellite cells, we performed an RNA-seq analysis on HDAC4 KO satellite cells in growth conditions. We found that most of the genes were up-regulated in the absence of HDAC4 in satellite cells, which is consistent with the role of HDAC4 as a transcriptional repressor. We continued the analyses on the genes that regulate skeletal muscle biology, among which the cyclin-dependent kinase inhibitor P21. This protein renders cell cycle withdrawal irreversible, inducing terminal differentiation of myoblast^8^. Although P21 was already known as a target of HDAC4 in cancer cells^62,63^, we revealed a new HDAC4-P21 axis in the regulation of satellite cell proliferation. Genetic ablation of P21 does not affect satellite cell number or proliferation^64^. However, when P21 expression was reduced in HDAC4 KO satellite cells by siRNA or UC2288 treatment, the number of satellite cells significantly increased, while satellite cell differentiation and fusion were unaffected. These results are very similar to the ones recently published for the arginine methyltransferase Prmt5 in adult stem cell^64^. Prtm5, indeed, regulates adult muscle stem cell expansion and differentiation, but not exclusively via epigenetic regulation of P21 gene. In fact, inhibition of P21 in Prmt5 mutant mice failed to rescue skeletal muscle regeneration^64^.

In addition, we identified Sharp1 as one of HDAC4 targets that may control satellite cell differentiation. It is a basic helix-loop-helix transcription factor that regulates important cellular processes in multiple cell types: from cell cycle exit to apoptosis, from tumor progression to circadian rhythms^65,66^. Sharp1 inhibits myogenic differentiation, via the lysine methyltransferase G9a, by interacting with MyoD and inhibiting its transcriptional activity^49,50,67^. Coherently, Sharp1-overexpressing muscle cells have reduced MyoD activity, resulting in impaired expression of differentiation genes, such as myogenin, MEF2 and MHC^49^. Moreover, Sharp1 null mice displayed enhanced differentiation of myogenic precursor cells in response to injury and satellite cell counts were not different from control mice. and satellite cell counts were not different from control mice^68^. It has been reported that Sharp1 expression is modulated by HDAC4 in fibroblasts^69^. However, the functional role of Sharp 1 in HDAC4-mediated inhibition of satellite cell differentiation was unknown. By repressing Sharp1 expression, HDAC4 allows satellite cells to fully differentiate. Indeed, down-regulation of Sharp1 expression in HDAC4 KO satellite cells leads to higher differentiation and fusion indexes, indicating that HDAC4 mediates satellite cell differentiation by regulating Sharp1 transcription. Interestingly, HDAC4 KO satellite cell number was unaffected by Sharp1 shRNA, demonstrating that HDAC4 regulates muscle cell proliferation and differentiation through multiple targets.

Our findings are important in light of the fact that pan-HDAC inhibitors (HDACi) are in use as therapeutic approaches to improve muscle regeneration in muscular dystrophies^70^. It has been demonstrated that fibro/adipogenic progenitors are the direct targets of HDACi in skeletal muscle^71^. However, systemic delivery of HDACi could indirectly affect HDAC4 target genes, whose inhibition is deleterious for satellite cell differentiation. Moreover, HDACs can either stimulate or block myogenesis in satellite cells, depending on the stage of myogenesis and the specific interactions between HDACs and other co-factors^72^.

To conclude, we demonstrated that HDAC4 regulates satellite cell proliferation, by repressing P21 expression, and satellite cell differentiation and fusion, by inhibiting Sharp1 expression. The identification of HDAC4 specific targets in satellite cells, provides a step forward in the development of new pharmaceutical approaches, in combination with HDACi, to improve muscle regeneration.

## Methods

### Mice

Mice were treated in strict accordance with the guidelines of the Institutional Animal Care and Use Committee, and relevant national and European legislation, throughout the experiments. Animal protocols were approved by the Italian Ministry of Health (authorization # 244/2013-B). HDAC4^fl/fl^ Pax7^CE/+^ Cre mice were generated by crossing HDAC4^fl/fl^ mice^34^ with Pax7^CE/+^ Cre mice, a mouse line expressing Cre recombinase under the control of Pax7 promoter, upon tamoxifen treatment^35^. In the HDAC4^fl/fl^ Pax7^CE/+^ mice, Cre gene is fused with the estrogen receptor domain and the ensuing protein, CreER, is confined to the cytoplasm. After tamoxifen (TMX) administration, CreER translocates into the nucleus and catalyzes the recombination of the loxP sites in HDAC4 gene, resulting in a frame-shift mutation. TMX (Sigma-Aldrich) was prepared as described in^35^ and [0.1 mg/g of body weight] injected intraperitoneally on a daily basis in 3-week-old mice, for 5 days.

### Freeze injury

Freeze injury was performed in eight-week old mice. The tip of a steel probe precooled in dry ice was applied to the tibialis anterior muscles of anesthetized mice for 10 seconds. This procedure induces a focal, reproducible, injury extending distally from the spike of the tibia and spreading over approximately one-third of the muscle as described in^47^.

### Histological analyses

TA muscles were dissected, embedded in tissue freezing medium (Leica, Wetzlar, Germany) and frozen in isopentane pre-cooled with liquid nitrogen. Cryosections (8 μm) were obtained by using a Leica cryostat. Hematoxylin and eosin (H&E) staining was performed according to the Sigma-Aldrich manufacturer’s instructions.

### Morphometric analyses

Myofiber cross-sectional area was quantified on H&E sections, by using Image J software.

### Satellite cell isolation by FACS

Satellite cells were isolated from skeletal muscles of 4-week-old mice by FACS, after enzymatic dissociation with a solution of CMF containing 0.02% BSA (Bovine Serum Albumin) (Sigma-Aldrich), 1% of Penicillin/Streptomycin (Sigma-Aldrich), 0.25 mg/ml Dispase II (Roche); 2 μg/μl Collagenase A (Roche), 8 mM CaCl_2_,10 ng/μl DNase I (Roche) and 5 mM MgCl_2_ for 2 hours at 37 °C. Cell suspensions were filtered through a 100-μm and a 40-μm nylon filters (Falcon) and stained with primary antibodies for 30 min on ice. The following antibodies were used: CD45- eFluor450 (eBioscience), Ter119- eFluor450 (eBioscience), CD31- Pacific Blue (Invitrogen) and Sca1-FITC (BD Bioscience), integrin- α7–APC (Ab-Lab). Flow cytometry analysis and cell sorting were performed on a DAKO-Cytomation MoFlo High Speed Sorter.

### Satellite cell isolation by GentleMACS

Satellite cells were isolated from 4-week-old mice also by using MACS microbeads technology (GentleMACS, Miltenyi Biotec, Bergish Gladbach, Germany). GentleMACS dissociation was performed according to the manufacturer’s protocol. Briefly, skeletal muscle was cut into small fragments and put in a C-tube with the enzyme cocktail of the dissociation Kit (# 130-098-305), according to the manufacturer’s recommendation. Muscles were then subjected to a mechanical disaggregation step in the GentleMACS dissociator, with an incubation at 37 °C. After dissociation, samples were filtered to remove any remaining larger particles from the single-cell suspension. Satellite cells were then isolated using the Satellite Cell Isolation Kit (#130-104-268), by depletion of non-target cells, which are magnetically labeled with a cocktail of monoclonal antibodies conjugated with MACS MicroBeads. Subsequently, a further step was performed to enrich satellite cells by using the anti-Integrin α-7 MicroBeads (#130-104-261).

### Culture conditions and treatments

C2C12 cells were grown in Dulbecco’s modified Eagle medium supplemented with 20% fetal bovine serum (Sigma-Aldrich), 2 mM glutamine (Sigma-Aldrich), 50 μg/ml gentamicin (Sigma-Aldrich) (GM). After one day, the medium was replaced with differentiation medium (DM) made of Dulbecco’s modified Eagle medium supplemented with 2% horse serum (Sigma-Aldrich), 2 mM glutamine (Sigma-Aldrich), 50 μg/ml gentamicin (Sigma-Aldrich).

Satellite cells were plated on 0,01% collagen (Sigma-Aldrich)-coated dishes with Dulbecco’s modified Eagle medium supplemented with 20% horse serum (Sigma-Aldrich),100 U/ml penicillin (Sigma-Aldrich), 100 μg/ml streptomycin (Sigma- Aldrich), 50 μg/ml gentamicin (Sigma-Aldrich), 3% of chicken embryo extract as growing medium (GM). After 3 days, the medium was replaced with differentiation medium (DM) (GM diluted 1:10).

To obtain HDAC4 deletion *in vitro*, satellite cells derived from HDAC4^fl/fl^ Pax7^CE/+^Cre+ mice were treated with 0.4 µM 4 OH-TMX (Calbiochem) or vehicle (methanol) as controls, for 72 hours, as described in^35^.

To inhibit P21 expression, satellite cells were treated with 10 µM UC2288 (trans-1-(4-chloro-3-trifluoromethyl-phenyl)-3-(4-hydroxy-cyclohexyl)-urea; Calbiochem) or vehicle (DMSO, Sigma-Aldrich), for 24 h hours, as described in^51^.

### Transfection of satellite cells

For transient transfection, 4 × 10^5^ HDAC4 KO satellite cells were seeded in 35-mm diameter plates. After 1 hour, cells were transfected with 4 µg of pSNAP-GFP combined with pLKO.1-puro Non-Target shRNA Control Plasmid DNA (Sigma-Aldrich) or Sharp1 shRNA (NM_024469_TRCN000086556, Sigma-Aldrich) in a 3:1 ratio, using Lipofectamine transfection reagent (Invitrogen), following the manufacturer’s instructions, except for the replacement of the medium 6 hours after transfection. Transfected cells were cultured in GM for 24 hours and then transferred to DM for additional 48 hours.

For siRNA transfection, we used the jetPRIME transfection reagent following manufacturer’s instructions. Briefly, 10 × 10^5^ HDAC4 KO satellite cells were seeded in 35-mm diameter dishes. The following day, cells were transfected with12 μl of 20 μM ON-TARGETplus Mouse Cdkn1a siRNA – SMARTpool, or ON-TARGETplus Non-targeting Control Pool (Dharmacon) as control, diluted in jetPRIME buffer and then added to jetPRIME reagent.

### Transduction of myotubes

Satellite cells (1,6 × 10^5^ cells/well) were seeded in 60-well plates and induced to differentiate. After 3 days in DM, 10^8^ TU/ml AV[Exp]-CMV>EGFP or AV[Exp]-CMV>-HDAC4.3SA-FLAG (Cliniscience) were added in 2 ml of DM. After 48 hours, GFP-fluorescence was observed to evaluate the effectiveness of viral transduction; cells were washed with PBS and total RNA was isolated. Addgene plasmid pcDNA-HDAC4.3SA-FLAG # 30486 was created by Tso-Pang Yao.

### Immunostaining analyses

For MHC immunofluorescence, differentiated cells were fixed in 4% PFA buffered solution for 10 minutes and then blocked with 10% goat serum in PBS for 1 hour. Cells were then incubated overnight with 1:10 sarcomeric MHC antibody (clone MF 20, Developmental Studies Hybridoma Bank) in 1% BSA PBS. To detect the primary antibody, we used a fluor-conjugated secondary antibody, anti-mouse IgG1 (Alexa488 or Alexa555, Invitrogen), diluted 1:500 in 1% BSA PBS. For α-sarcomeric actin immunofluorescence, cells were fixed in 4% PFA buffered solution for 10 minutes and permeabilized in 0.1% Triton-X 100 (Sigma-Aldrich). Cells were then incubated for 1 hour with 1:100 monoclonal anti-Actin (α-Sarcomeric) antibody (Sigma-Aldrich) in PBS. Fluor-conjugated anti-mouse IgG1 secondary antibody (Alexa 555, Invitrogen) diluted 1:500 in 1% BSA PBS was used to detect the primary antibody.

For Pax7, cells were fixed in 4% PFA buffered solution for 20 minutes at room temperature and permeabilized with absolute methanol for 6 minutes at −20 °C. Then, cells were heat-activated with 0.01 M of citrate buffer pH 6.0 for 10 minutes. Samples were then blocked for three hours with 4% BSA in PBS and incubated overnight with 1:20 anti-Pax7 antibody (mouse monoclonal, Developmental Studies Hybridoma Bank). After washing in 0.1% BSA/PBS, cells were incubated with 1:1000 biotin-conjugated secondary antibody (Jackson ImmunoResearch) for 45 minutes at room temperature. Then the samples were incubated with 1:2500 streptavidin antibody (Jackson ImmunoResearch) for 30 minutes at room temperature.

To better detect the green fluorescent protein in transfected cells, we used primary anti-GFP antibody (Life technologies) diluted 1:400 in 1% BSA PBS, by following the above protocol and adding a permeabilization step of 5 minutes with 0.25% Triton-X 100 (Sigma-Aldrich) in PBS before blocking in 10% goat serum in PBS.

For P21 immunofluorescence, cells were fixed with 4% PFA buffered solution for 10 minutes and permeabilized with 0.25% Triton-X 100 (Sigma-Aldrich). After 1 hourin 5% BSA PBS blocking solution, cells were incubated overnight with 1:100 p21 Waf1/Cip1 monoclonal antibody (Cell Signaling) in 1% BSA PBS. Fluor-conjugated anti-rabbit IgG1 secondary antibody (Alexa568, Invitrogen) diluted 1:500 in 1% BSA PBS was used to detect the primary antibody. Nuclei were counterstained with 0.5 μg/ml Hoechst and samples were mounted with 60% glycerol in Tris HCl 0.2 M pH 9.3.

### BrdU assay

Satellite cells were treated with 50 μM BrdU (Fluka® Analytical) for 1 hour, then fixed with 5% acetic acid and 95% ethyl alcohol for 20 minutes and incubated with HCl 1.5 M for 5 minutes at room temperature. After permeabilization with 100% methanol for 6 minutes at −20 °C, cells were blocked with 5% goat serum in PBS for two hours at room temperature and incubated with anti-BrdU primary antibody (AbD Serotec), diluted 1:50 in 4% BSA, overnight at 4 °C. After being washed in PBS, cells were incubated for 1 hour at room temperature with goat anti-rat secondary antibodies (Alexa488, Invitrogen), diluted 1:200 in 1% BSA in PBS.

### Tunel assay

Tunel assay was performed using the ApopTag® Fluorescein *In Situ* Apoptosis Detection Kit (Merck Millipore) following the instructions from the manufacturer. Positive controls were obtained by treating satellite cells with 1 μg/mL DNase I for 10 minutes at room temperature.

### Equipment and settings

Images were acquired using an Axio imager A2 system equipped with an Axiocam HRc, and Axiovision Release 4.8.2 software (Zeiss, Oberkochem, Germany), at standard resolution (1300 × 1030 pixel).

### Protein extraction and western blot analyses

Satellite cells were washed and collected in 100 μl of lysis buffer (50 mM Tris HCl pH 7.4, 1 mM EDTA, 150 Mm NaCl, 1%Triton) supplemented with protease and phosphatase inhibitors. After 30 minutes of incubation, cells were centrifuged and supernatant were frozen. Cell lysates were concentrated by SpeedVac. Proteins (10–20 micrograms) were separated by SDS-PAGE and transferred to PVDF membrane (Invitrogen). Unspecific binding was blocked with 5% nonfat dry milk in TBST buffer (20 mM Tris HCl pH 7.6, 137 mM NaCl, 0.5% Tween 20) and then membranes were incubated overnight, at 4 °C, with primary antibody diluted in 5% BSA (Sigma-Aldrich) in TBST. After washing in TBST, membranes were incubated with HRP secondary antibodies conjugates (BIO-RAD 170-6515 or 170-6516) and signals were detected by using ECL chemistry (Cyanagen). Images were acquired on ChemiDoc MP imaging system (BIORAD) with Image Lab 5.2.1 software.

The following primary antibodies were used: anti-α-Tubulin (Sigma-Aldrich), HDAC4 (Santa Cruz); MHC (MF20, Developmental Studies Hybridoma Bank).

### RT-PCR and real-time PCR

Total RNA from satellite cells was isolated using TRIzoL reagent (Thermofisher), according to manufacturer’s instructions. cDNA synthesis was performed using Reverse Transcription Kit (Takara) from 0.5–1 μg of RNA, following manufacturer’s instructions. Quantitative PCR was performed using the ABI PRISM 700 SDS (Applied Biosystems) with SYBR Green reagent (Applied Biosystems) and primer listed in Supplementary Table S2.

### RNA-seq

Total RNA collected from satellite cells, after 24 hours in growing conditions, was purified by using RNeasy MinElute Cleanup Kit (Qiagen). About 0.8 μg of RNA from duplicates was subjected to transcriptome analysis at the NextGen Sequencing facility (IGA Technologies Service, Udine). Name of the organism that is the target of sequencing: Mus musculus. “TruSeq Stranded mRNA Sample Prep kit” (Illumina, San Diego, CA) was used for library preparation. Libraries were processed with Illumina cBot for cluster generation on the flow cell and sequenced on single-end mode at the multiplexing level requested on HiSeq. 2500 (Illumina, San Diego, CA). The Illumina pipeline based on CASAVA version 1.8.2 of the was used to process raw data for both format conversion and de-multiplexing. Standard bioinformatics analysis was provided by the IGA facility. Briefly, the reads produced (on average 56.4 M reads/sample; min 47.6 M, max 76.8 M) were first put through the sequencing pipeline made of base-calling on the Illumina Pipeline, trimming (min length 35) with ERNE^73^ and removal of adapter sequences with Cutadapt. Reads were then aligned to GRCm38 reference genome/transcriptome using TopHat^74,75^. Overall alignment rate was 95% for all the samples. Cuffdiff was used to perform differential expression analysis^76^. Additional bioinformatics analyses of the RNA-seq results were performed by using available online tools for gene ontology (http://www.pantherdb.org/). Validation of RNA-seq results was done on independent samples by real-time PCR. Data were uploaded in GEO (reference Series number: GSE106485).

### Statistical Analyses

Data are presented as mean ± SEM of independent biological replicates as indicated in each figure. Statistical significance was determined using two-tailed Student’s t-test with significance defined as p < 0.05 (*), p < 0.05 (**) and p < 0.001 (***).

## Electronic supplementary material

Supplementary information
