# Supplementary material for: HDAC4 regulates satellite cell proliferation and differentiation by targeting P21 and Sharp1 genes
Source: Sci Rep. 2018 Feb 22;8:3448. doi: 10.1038/s41598-018-21835-7 (PMC5823886; doi:10.1038/s41598-018-21835-7)
Supplement: Supplementary file 1 — Supplementary information [file 41598_2018_21835_MOESM1_ESM.pdf]

HDAC4 regulates satellite cell proliferation and differentiation by targeting P21 and Sharp1 genes

Nicoletta Marroncelli[1], Marzia Bianchi[1], Marco Bertin[1], Silvia Consalvi[2], Valentina Saccone[2], Marco De Bardi[2], Pier Lorenzo Puri[2], Daniela Palacios[2], Sergio Adamo[1], Viviana Moresi[1,3,*]

Supplementary Information

Supplementary Figure Legends

Supplementary Figure S1. HDAC4 expression is reduced in HDAC4 KO satellite cells. HDAC4 expression in HDAC4 KO and control satellite cells, analyzed by (**a**) real-time PCR (n=6 each sample) and (**b**) western blot (cropped blots) analyses, with relative densitometry (n=3 each sample). α-Tubulin was used as a loading control. Full-length blots are presented in Supplementary Figure S2A. Data are presented as mean ± SEM. *p<0.05; **p<0.005 (Student's t-test).

Supplementary Figure S2. Full-length western blots. Uncropped gels showing the expression of (**a**) HDAC4 and (**b**) MHC in HDAC4 KO (KO) and control (C) satellite cells. α-Tubulin was used as a loading control.

Supplementary Figure S3. HDAC4 intrinsically regulate satellite cell differentiation. (**a**) Immunofluorescence for MHC in wild-type satellite cells cultured with conditioned medium from HDAC4 KO or control satellite cells. Conditioned media from n=3 mice, per each condition. Scale bar: 100 µm. (**b**) Quantification of the differentiation and fusion indexes in wild-type satellite cells cultured with conditioned medium from HDAC4 KO or control satellite cells. n=3 each condition. Data are presented as mean ± SEM. (**c**) HDAC4 expression in satellite cells treated with vehicle or 4OH-TMX, analyzed by real-time PCR. n=4 each sample. Data are presented as mean ± SEM. *p<0.05 (Student's t-test).

Supplementary Figure S4. HDAC4 does not affect apoptosis in satellite cells. (**a**) Tunel assay and quantification of apoptotic cells in control, HDAC4 KO samples, and positive controls of the assay. Scale bar: 50 and 100 µm. n=5 per genotype; n=3 for the positive controls. **p<0.005 (Student's t-test). (**b**) Expression of indicated apoptotic markers in control and HDAC4 KO satellite cells. n=4 for each sample. Data are presented as mean ± SEM.

Supplementary Figure S5. Overexpression of HDAC4 in HDAC4 S/A overexpressing muscle cells. HDAC4 expression in primary myotubes infected with adenovirus expressing HDAC4 S/A or GFP, as control. n=5 GFP, n=7 HDAC4 S/A. Data are presented as mean ± SEM. ***p<0.001 (Student's t-test).

Supplementary Figure S6. Downregulation of P21 in HDAC4 KO satellite cells. (**a**) Immunofluorescence for P21 in HDAC4 KO satellite cells treated with UC2288 or

vehicle for 24 hours. Scale bar: 50 µm. (**b**) Expression of P21 in HDAC4 KO satellite cells transfected with a pool of four siRNA against P21 (P21) or a pool of non-targeting siRNA (Scr), as control. n=5 Scr and n=7 P21. Data are presented as mean ± SEM. *p<0.05 (Student's t-test).

Supplementary Figure S7. Downregulation of Sharp1 in muscle cells. Sharp1 expression in C2C12 cells transfected with Sharp1 shRNA or scramble (shScr) expression vector, as control. n=4 each sample. Data are presented as mean ± SEM. *p<0.05 (Student's t-test).

Supplementary Table S1. List of genes in each gene ontology category of the RNA-seq results.

Biological Process	Genes
Response to stimulus	fas, taok1, fap, ccl2, srpk3, hmga2, fhl1, hspb3, dact2, pdgfb, mapkapk3, f3, spint1, ctsh, sema3c, nod1, eva1c, penk, slit2, cxcl5, lif, fbn1, ndrg1, hk1, ngf, abcc9, map3k2, itgb7, igha, ptgir, gabre, cgref1, sema3e, anxa8, gpx3, cxcl3, ttc1, itgb5, il20rb, tie1, txndc2, hspb8, gpx3, plet1, mlxipl, gpr97, tnfaip3, wnt11, pak6, csf1, gria3, nkd2, tll1, pamr1, pdk1, slc30a4, inhba, pik3c2a, trp53inp1, arhgap23, slc39a4, tnfrsf1b, atg9b, tshr, cryab, gpx7, sqstm1, clec4d, ubxn2b, xiap, ptk2b, gal, sema7a, tek, kpna3, crip1, angpt4, anpep, wnt5a, cma1, il12rb2, fzd6, ccl9, bdkrb1, zfp385a, cxcl2, gpr37l1, cd55, p2rx6, sod3, mapk13, procr, icam1, ppp3cb, tfpi2, hspb7, neto2, cyr61, rcan2, timp3, pik3r3, crlf1, cdc42ep1, ppap2b, cxcl14, hmga1 , tec, klk8, il11, adrb2, def6, rom1, nuak2, ccl7, phldb3, fcgrt, il21r, fignl1, pdzd2, ndrg2, sgk1, osmr, blnk, itgb3, efna5, cxcl16, wnt10b, ikbke, hspb1, cx3cl1, eda2r, pgf, p2rx7, klk8, ncf2, itpr3, ttc12, rad1, wnt7b, psme1, tec, trib3, hfe2, adat1, ctgf, dusp6, mocos, tmx3, slc38a3, ngfr, il4ra, cd99
Developmental process	scube1, fas, taok1, mier3, fgf1, plxna4, numbl, myo7a, lce1g, csrp3, bmpr2, fhl1, mbp, micall1, mef2c, slit2, phex, fgf11, cdh5, cidea, fgf5, vat1, mical2, fbn1, ngf, cdh19, myh11, adamts1, tpm1, map3k2, lmod2, notch4, traf1, fubp1, olfml2a, fgd3, lce1f, tie1, tmeff2, dusp1, rbp1, maob, spdef, phactr1, plet1, myh3, gpr97, wnt11, pak6, foxo3, cgnl1, csf1, dtx4, tll1, b3galt5, inhba, crabp1, tnfrsf1b, ldb3, tshr, myh9, myh8, pacsin1, clec3b, neurl3, adam3, xiap, ptk2b, pvalb, arhgef28, col4a1, lama2, tmp3-rs7, bhlhe22, tek, ier3, crip1, arhgef3, angpt4, wnt5a, tnc, myom2, il12rb2, myf6, fabp4, itm2b, cd80, bhlhe40, col16a1, zfp385a, mbnl2, gpr37l1, myl2, mybpc2, ptgis, ntn4, olfml3, tgfb1i1, ppp3cb, neto2, cyr61, pcdh7, cdh11, syne4, plekhb1, tlr9, ankrd2, map6, tec, klk8, phldb3, mcam, il21r, acp5, plxnb3, myod1, fmod, intm2c, utrn, pdzd2, neurl3, dmp1, ndrg2, fat1, mylpf, vdr, sgk1, osmr, tnmd, lamc3, efna5, myo1e, atoh8, wt10b, scube3 adam12, lingo2, pcdh20, myh7, crabp2, foxd3, eda2r, pgf, gli2, creb3l1, jag2, clip1, lce1h, gdnf, wnt7b, tec, lrrn1, adat1, plp1, ctgf, dusp6, myog, hoxa10, bhlhe41, ngfr, mybpc1, hoxb2, col5a3, tgfbr1
Metabolic process	isg15, elovl6, rpl39l, cyb5a, mier3, hk1, aebp1, rbpj, h2afj, ak1, plxna4, lce1g, fap, ccl2, hmga2, fhl1, larp7, hspb3, pddc1, ass1, anxa5, anxa3, apobec1, g2e3, upf3a, ube3a, mapkapk3, hist1h1c, spint1, pck2, fbp2, ctsh, selt, adh7, ptma, pdk1, cox6a2, aim1, nod1, atp9a, ptms, gfpt2, phex, naa50, hmgcs1, gm20939, ppp1r3c, psmc1, ndufs7, cpeb2, ugcg, prrg1, pcna-ps2, mt-nd2, adamts1, enpp1, hebp2, slc1a2, igha, tbc1d2, pgam2, s100a16, ptpre, cpt1c, adprhl1,

	fas, srsf7, fubp1, olfml2a, atp11b, lce1f, adamtsl5, anxa8, has2, gpx3, azin2, reep2, mterf1b, cyp4f17, ttc1, ucp1, sh3kbp1, ptges, hdac4, plin2, txndc2, maob, pim3, acox2, cast, spdef, hspb8, plin1, manba, ptprf, gpx8, zc3hav1l, ctdspl, slc5a5, mlxipl, pmm1, tnfaip3, pak6, grhl3, tfap4, khk, dctd, rgs10, foxo3, hist1h2bc, mbp, plau, cbx3, hccs, dtx4, zik1, mvp, xrcc5, pamr1, pdk1, seh1l, galnt6, ptgr1, sp3, b3galt5, phf6, cox8b, inhba, pik3c2a, ptpn7, trp53inp1, arhgap23, lox, rnf138, sp4, six4, cyp26b1, ptp4a3, osr1, fap, mdx1, prodh2, s100a7a, atg9b, nrap, hebp1, ldb3, tshr, mdx4, cryab, srsf10, mgam, adamtsl2, gpx7, bmi1, ttll9, pcyox1l, slpi, sqstm1, ubxn2b, pknox1, xiap, plin5, ptk2b, alg6, capn3, gm10073, bhlhe22, hist3h2ba, mov10l1, aim1, ppp1r27, prkcsh, rybpcrip1, klhdc7a, anpep, cma1, ndufaf2, rps27, ctsw, myf6, abca1, ccl9, mt-nd4, itm2b, atp10a, atad2, gm10330, ythdc2, galm, xdh, ddx3y, pura, zfp385a, adamtsl1, sox2, sulf1, s100a8, suynm, impad1, calr3, agt, loxl4, gpr37l1, rgs3, mt-co1, trove2, ptgis, olfml3, cpa4, cnot6, zfp418, cstb, pif1, pck1, sp7, gm8730, ppp3cb, tfpi2, hspb7, gar1, acsbg1, cav3, tcap, rcan2, kif1a, etnk1, mdm2, timp3, pik3r3, ankrd2, ucp2, cdc42ep1, alox5, ppap2b, hmga1, nedd8, st18, las1l, adrb2, dcun1d1, ddx6, shpk, def6, ptpn13, gt1, ampd3, nuak2, ccl7, ptprv, pcyt1b, ckb, eef1a2, fignl1, uty, asprv1, loxl1, thap2, s100a9, aff3, plxnb3, cwc15, clock, lsm8, pphln1, atp6v1h, e2f2, myod1, fmod, gpd1l, mettl4, gtf2f1, gpd1l, itm2c, sox10, stk17b, sox11, dmp1, adamtsl3, hcls1, sord, metap2, tbc1d9, htra3, sulf2, klhl26, sgk1, zfp81, larp4, slc1a5, id3, rgs16, atoh8 hr, ampd1, lingo2, nucks1, gss, d7ertd443e, hspb1, peak1, rpl19, kbtbd7, rgs9, slc25a48, cx3cl1, cpe, polr2f, tbca, sult4a1, foxd3, mt-nd5, pgm5, eda2r, leo1, trmt61a, sln, klk8, syf2, ptges3, pla2g4a, itih5, os9, clip1, lce1h, ttc12, rad1, purb, hexa, cpox, urb1, atp13a3, ctrb1, psme1, bdh1, tec, parp14, slc27a1, zbtb14, trib3, lmcd1, ppif, sacm1l, ero1l, nt5dc2, lrrn1, tescl, hfe2, pias3, uchl1, rnf19b, adat1, atp2a1, myog, lpin1, hoxa10, zfp28, hmox1, fmr1, eif2s3y, arhgap5, hsd3b6, hoxb2, mt-atp8, atp2b4, pqlc3
Cellular process	scube1, tbkbp1, fas, taok1, tnfaip2, elovl6, rpl39l, spink2, mier3, arhgdib, hk1, aebp1, actg2, fgf1, h2afj, plxna4, numbl, myo7a, cotl1, lce1g, fap, gprc5a, ccl2, srpk3, tmem202, gng11, hmga2, pddc1, daam2, ass1, dact2, mbp, capg, pdgfb, mapre3, eube3a, mapkapk3, hist1h1c, cd36, ctsh, pdk1, pls3, rap1a, synm, atp9a, palm3, eva1c, scn1b, penk, mgarp, mgmt1, slit2, cxcl5, gfpt2, phex, fgf11, ccnd2, slco2a1, cidea, lif, mzt1, mpz, rbp1, orai2, fgf5, naa50, gm20939, fbn1, akap2, ndrg1, hk1, psmc1, cpeb2, ugcg, ngf, caq1, appl1, slc4a7, tnxb, pcna-ps2, myh11, adamts1, tmp1, zfyve1, stx16, map3k2, lmod2, itgb7, slc1a2, igha, tbc1d2, pgam2, s100a16, adprhl1, traf1, fubp1, olfml2a, atp11b, clta, lce1f, adamtsl5, has2, azin2, mterf1b, aatk, cxcl3, arhgap36, cltb, itgb5, ucp1,

	tmeff2, dusp1, hdac4, txndc2, rab6b, maob, acox2, spdef, manba, sesn2, phactr1, mthfd2, kcnj4, dock1, sat1, plet1, ctdspl, gjb5, myh3, slc5a5, mlxipl, gpr97, tnfaip3, cald1, gjc3, wnt11, pak6, grhl3, tfap4, rgs10, snx30, foxo3, cgnl1, csf1, hist1h2bc, stx11, mbp, gria3, rnd1, nkd2, vamp7, cbx3, rasl11a, dtx4, zik1, tll1, mvp, xrcc5, pde1a, nefm, map7, tnfaip6, pdk1, slc30a4, sp3, b3galt5, cox8b, inhba, fastkd1, pik3c2a, trp53inp1, arhgap23, slc39a4, plk2lox, rnf138, sp4, nes, aplp1, pde8a, tnfrsf1b, vat1, nedd1, ptp4a3, fap, gpr176, s100a7a, atg9b, nrap, ldb3, tshr, slc18a2, myh8, pacsin1, pcyox1l, sqstm1, il1rl1, clec4d, neurl3, ubxn2b, xiap, otoa, map7d2, ptk2b, gal, alg6, pds5b, arhgef28, capn3, bfsp1, col4a1, lama2, espn, tpm3-rs7, gm10073, hist3h2ba, plek2, mov10l1, aim1, kpna3, ccdc80, ier3, arhgef3, klhdc7a, anpep, wdr35, wnt5a, tnc, actc1, myom2, emp2, ndufaf2, kcnf1, rps27, crim1, ctsw, myf6, abca1, ccl9, itm2b, atp10a, atad2, cck, ythdc2, xdh, krt19, apbb1ip, bdkrb1, ddx3y, gsn, col16a1, zfp385a, adamtsl1, cxcl2, mdk, arl5a, s100a8, impad1, agt, loxl4, gpr37l1, cd55, rgs3, mybpc2, ptgis, gem, ntn4, olfml3, tgfb1i1, sparcl1, p2rx6, sfn, sod3, mapk13, procr, tecta, zfp418, icam1, pif1, sp7gm8730, ppp3cb, cyr61, fxyd5, tubb2a, syne4, plekhb1, cav3, tcap, rcan2, kif1a, ptn, mdm2, tlr9, atp1b2, timp3, pik3r3, ankrd2, ucp2, rgl1, cdc42ep1, map6, ppap2b, cxcl14, hmga1, cdkn1b, acta2, casq2, sncg, las1l, tec, klk8, il11, btg2, adrb2, dcun1d1, cdkn1a, rhobtb3, dmp1, ddx6, shpk, rom1, tgfbi, nuak2, alrl8a, ccl7, phldb3, mcam, gpnmb, fcgrt, il21r, krt76, eef1a2, fignl1, itgb1bp2, loxl1, s100a9, aff3, plxnb3, lsm8, e2f2, myod1, fmod, mettl4, itm2c, stk17b, neurl3, dmp1, adamtsl3, hcls1, ndrg2, sept1, tbc1d9, grb14, htra3, klhl26, vdr, rap2c, sgk1, abcb7, nup62cl, mob1b, blnk, lamc3, itgb3, slc1a5, efna5, rab32, myo1e, sphk1, rgs16, cxcl16, wnt10b, scube3, map1a, lingo2, tgfbr3, nucks1, ikbke, tubb2b, plscr2, pls3, slc16a1, tubb4a, peak1, depdc1a, pde8b, rpl19, kbtbd7, myh7, cacna1s, arap2, rgs9, fxyd3, slc25a48, cx3cl1, cpe, polr2f, lin7c, ccdc109b, foxd3, cd34, pgm5, eda2r, leo1, pde1b, pgf, islr, jsrp1, creb3l1, trmt61a, p2rx7, sln, syf2, krt8, camk1d, des, os9, itpr3, lfng, clip1, lce1h, slc4a11, vps11, gdnf, wnt7b, atp13a3, tec, agtr2, zbtb14, trib3, cldn5, lmcd1, ppif, sacm1l, nt5dc2, lrrn1, tro, tescl, rhobtb2, hfe2, 9530053a07rik, uchl1, rnf19b, adat1, plp1, ctgf, chordc1, lgals9, atp2a1, dusp6, myog, lpin1, tmx3, zpf28, trpv2, fmr1, kcna6, slc38a3, slc5a3, eif2s3y, ngfr, mybpc1, il4ra, kcnj8, atp2b4, svop

Supplementary Table S2. Primer used for real-time PCR

Gene	Primer Forward	Primer Reverse
HDAC4	GTCTTGGGAATGTACGACGC	GTTGCCAGAGCTGCTATTTG
Cyclin E1	GGCGAGGATGAGAGCAGTTC	CGATCAAAGAAGAAGTCCTGTGCC
Cyclin A2	CCCTGCATTTGGGTGTGAAC	GGTGCTCCATTCTCAGAACC
Pax7	TCCCCCTGGAAGTGTCCA	TGGGAAACACGGAGCTGA
e-MHC	TGGTCGTAATCAGCAGCA	TCGTCTCGCTTTGGCAA
P21	AGCGCGTTCGGAGCCTA	CCGTTTTCGGCCCTGAGA
Sharp1	GGAGCTTGAAGCGAGACGAT	GAGCGCTCCCCATTCTGTAA
Bax	GCCTCCTCTCCTACTTCGGG	TGAGGACTCCAGCCACAAAGA
Bcl-2	AAGCTGTCACAGAGGGGCTA	TCTCAGGCTGGAAGGAGAAG

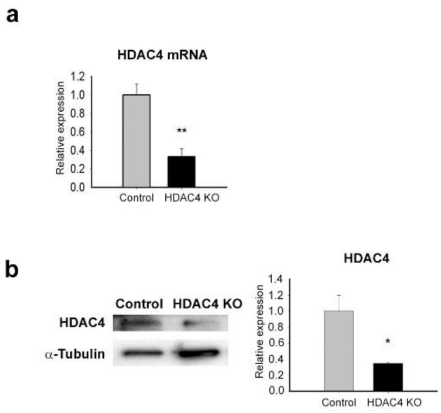
a
HDAC4 mRNA
Relative expression
1.2
1.0
0.8
0.6
0.4
0.2
0.0
**
Control
HDAC4 KO
b
Control
HDAC4 KO
HDAC4
α-Tubulin
HDAC4
Relative expression
1.4
1.2
1.0
0.8
0.6
0.4
0.2
0.0
*
Control
HDAC4 KO

a

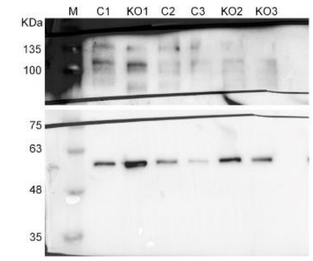
M
C1
KO1
C2
C3
KO2
KO3
KDa
135
100
75
63
48
35

b

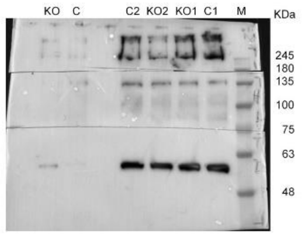
KO
C
C2
KO2
KO1
C1
M
KDa
245
180
135
100
75
63
48

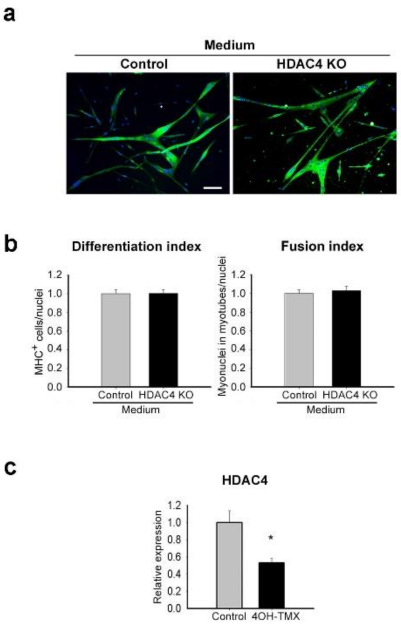
a
Medium
Control
HDAC4 KO
b
Differentiation index
MHC+ cells/nuclei
1.2
1.0
0.8
0.6
0.4
0.2
0.0
Control
HDAC4 KO
Medium
Fusion index
Myonuclei in myotubes/nuclei
1.2
1.0
0.8
0.6
0.4
0.2
0.0
Control
HDAC4 KO
Medium
c
HDAC4
Relative expression
1.2
1.0
0.8
0.6
0.4
0.2
0.0
*
Control
4OH-TMX

a

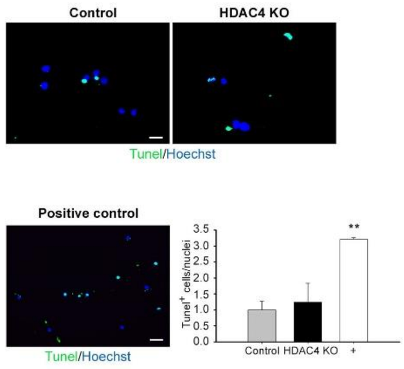
Control
HDAC4 KO
Tunel/Hoechst
Positive control
Tunel/Hoechst
Tunel+ cells/nuclei
3.5
3.0
2.5
2.0
1.5
1.0
0.5
0.0
**
Control
HDAC4 KO
+

b

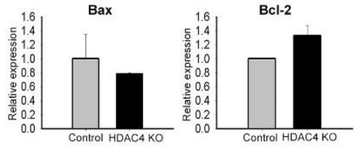
Bax
Relative expression
1.6
1.4
1.2
1.0
0.8
0.6
0.4
0.2
0.0
Control
HDAC4 KO
Bcl-2
Relative expression
1.6
1.4
1.2
1.0
0.8
0.6
0.4
0.2
0.0
Control
HDAC4 KO

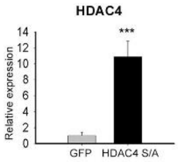
HDAC4
***
Relative expression
14
12
10
8
6
4
2
0
GFP
HDAC4 S/A

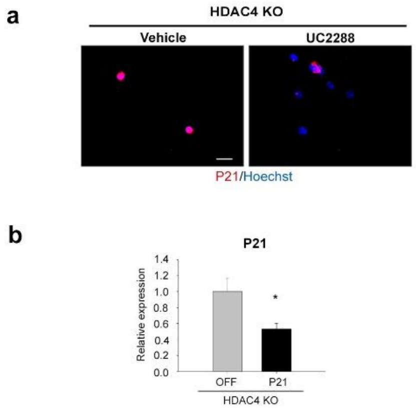
a
HDAC4 KO
Vehicle
UC2288
P21/Hoechst
b
P21
Relative expression
1.4
1.2
1.0
0.8
0.6
0.4
0.2
0.0
*
OFF
P21
HDAC4 KO

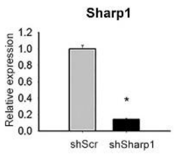
Sharp1
Relative expression
1.2
1.0
0.8
0.6
0.4
0.2
0.0
*
shScr
shSharp1
